# Implications of the impact of prevalence on test thresholds and outcomes: lessons from tuberculosis

**DOI:** 10.1186/1756-0500-5-563

**Published:** 2012-10-10

**Authors:** Tanya GK Bentley, Antonino Catanzaro, Theodore G Ganiats

**Affiliations:** 1 Director, Health Economics and Outcomes Research, Partnership for Health Analytic Research, LLC, 280 S Beverly Drive #404, Beverly Hills, CA 90212-3904, USA; 2 Professor, University of California San Diego, Department of Medicine, 9500 Gilman Drive #0643, La Jolla, CA 92093-0643, USA; 3 Executive Director, University of California San Diego, Health Services Research Center, 9500 Gilman Drive #0622, La Jolla, CA 92093-0622, USA

**Keywords:** Disease prevalence, Testing, Screening, Test thresholds, Sensitivity, Specificity, Tuberculosis, Outcomes

## Abstract

**Background:**

With today’s rapid advances in technology and understanding of disease, more screening and diagnostic tests have become available in a variety of sociodemographic and clinical settings. This analysis quantifies the impact of varying prevalence rates on test performance for given sensitivity and specificity values.

**Methods:**

Using a worked example of latent tuberculosis infection, we compared true-positive (TP) and false-positive (FP) results when varying prevalence and test sensitivity and specificity. We used estimates from published literature to estimate two tests’ sensitivity (81%, QuantiFERON^®^-TB Gold In-Tube; 88%, T-SPOT^®^.*TB*) and specificity (99%; 88%), and we used World Health Organization data to estimate disease prevalence in five countries.

**Results:**

Varying sensitivity impacted outcomes most in high-prevalence settings; change in specificity had greater impact in low-prevalence settings. In switching from QuantiFERON-TB to T-SPOT.*TB* (higher sensitivity, lower specificity), trade-offs between increasing case identification (TPs) and decreasing unnecessary treatments (FPs) varied dramatically with prevalence. Lower-prevalence settings paid a greater “price” of more FPs for each TP gained, with 37.7 FPs per TP in the United States (5% prevalence) versus 2.5 in the Ivory Coast (55% prevalence).

**Conclusions:**

Prevalence affects test performance for given sensitivity and specificity values. To optimize test performance, disease prevalence should be incorporated in testing decisions, and sensitivity and specificity should be set locally, not globally. In lower-prevalence settings, using highly specific assays may optimize outcomes.

## Background

With today’s rapid advances in technology and understanding of disease, more screening and diagnostic tests have become available in a variety of sociodemographic and clinical settings. Although these tests can be described in various ways (such as use of receiver operating characteristic curves and predictive values), a primary factor is the number of true-positive, false-positive, true-negative, and false-negative results that occur when implementing a test. In addition to various environmental factors, these outcomes are determined in large part by test sensitivity and specificity. For tests measured on a continuous scale (e.g., blood glucose, valued in mmol/L) and dichotomized to “normal” or “abnormal,” sensitivity and specificity are determined in large part by the positive test threshold: the test result that divides positively defined results from those considered negative.

The relative impact of sensitivity and specificity on true and false results and on disease outcomes is described in Table 1. Although in reality these associations are rarely so direct because such tests are not typically used in isolation (e.g., further diagnosis or confirmation of the condition often follows positive results), we simplify here for the purposes of demonstration only. Sensitive tests are used to accurately identify those *with* disease, and negative results from such tests are used to rule *out* disease. Specific tests are used to identify those *without* disease, and positive results are used to rule *in* disease. Highly sensitive tests therefore help detect disease and, in the case of infection, possibly reduce the chance of spread to others; highly specific tests, on the other hand, help prevent unnecessary treatments, toxicities, and costs among individuals *not* infected with disease.

**Table 1:** Potential outcomes of screening tests with varying levels of sensitivity and specificity

**Test characteristic**	**High sensitivity**	**High specificity**
Impact on results	↑ True positives	↑ True negatives
Testing goal	Identify people with disease	Identify people without disease
Treatment goal	· Treat disease	· Avoid unnecessary treatment
	· Prevent future illness and, in the case of infection, possible disease spread	
Potential harms of opposite test characteristic	*Low sensitivity:*	*Low specificity:*
	· ↑ False negatives	· ↑ False positives
	· ↑ Potential future illness and suffering	· ↑ Bodily harms, toxicity, and financial costs of unnecessary treatment
	· ↑ Potential future spread of disease (in the case of infection)	· ↑ Social stigmatization
		· ↓ confidence in screening program

A given test’s sensitivity and specificity are negatively associated: increasing sensitivity by changing the positive test threshold results in a decreased specificity, and vice versa. In this way, the selection of a positive test threshold involves an inherent balancing act. For example, increasing test sensitivity results in decreased specificity and leads to more true- and false-positive results. Increasing test specificity results in decreased *sensitivity* and leads to *fewer* true- and false-positive results. Thus, in seeking the sensitivity-specificity balance when determining the positive test threshold, a key question for clinicians and policymakers is how many false positives they are willing to tolerate in order to get one additional true positive.

The magnitude of this trade-off between true and false positives, the relative effects of sensitivity and specificity, and, thus, decisions regarding optimal thresholds are determined by multiple factors [1,2]. For example, there are local considerations of capacity, staffing, and clinician and technician skill; comorbidities, including immunosuppression; clinician and patient preferences; disease characteristics; and variations in health resources. In addition to these factors, disease prevalence plays a significant role.

We propose that disease prevalence must be considered in conjunction with other disease- and setting-specific factors when setting a test’s positive result threshold and when deciding between tests. Specifically, decisions made in low-prevalence settings must not be automatically extrapolated to settings with high disease prevalence.

**Figure 1:**
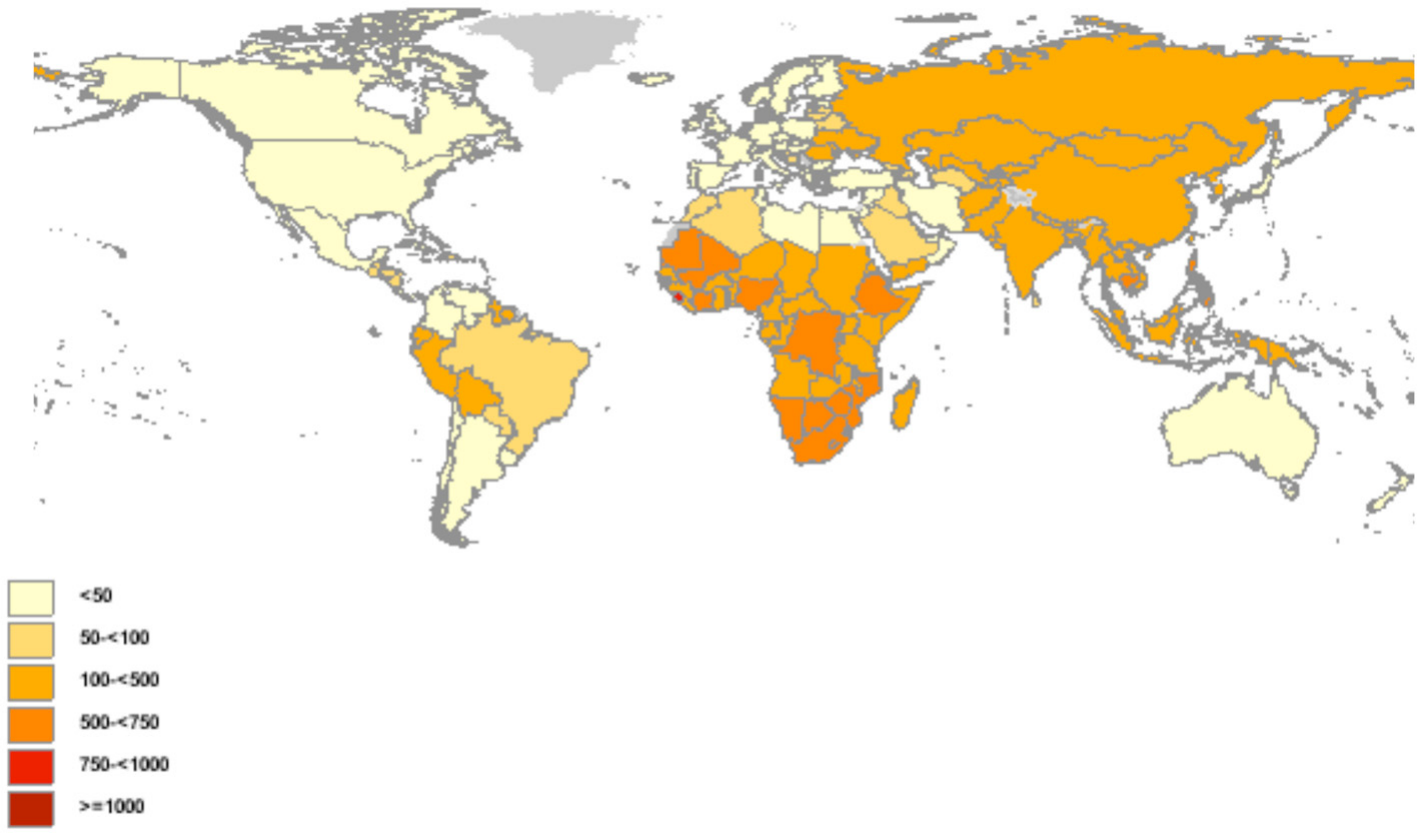
**Total TB prevalence per 100,000 populations by country, 2007. ***Source*: World Health Organization Global TB Database [4].

This issue is especially critical for diseases with widely varying prevalence and is thus well demonstrated with tuberculosis (TB). TB is a common airborne disease that primarily affects the lungs and infects over 2 billion people worldwide. Although 90% of those infected have latent, or inactive, tuberculosis infection (LTBI), one in ten will become ill with active TB, and close to 2 million will die from it each year. TB prevalence varies dramatically from country to country (Figure 1) [3]; the prevalence of LTBIs in 2006 was 5% in the US compared with 43% in Uganda and 55% in the Ivory Coast [4]. An estimated 80% of the 8.8 million new infections in 2005 were concentrated among 20 countries, and more than half of TB-related deaths in 2006 occurred in Asia [5]. In addition, although the per-capita incidence of TB is stable or falling in all six World Health Organization (WHO) regions, the number of cases and deaths continue to rise due to population growth [5].

For many years, the WHO’s only LTBI testing option has been the TB skin test, yet its implementation is challenging because of its greatly diminished sensitivity and specificity among certain populations [6]. For example, skin test sensitivity is reduced in individuals with immunosuppressive diseases such as HIV infection and among those taking immunosuppressant medications, and specificity is diminished in people who have had prior bacille Calmette-Guérin vaccine and in those infected with non-TB environmental bacteria [6-8].

New interferon–*γ* release assay (IGRA) tests have consequently been developed for diagnosing LTBI and offer improved operating characteristics over the standard skin test. However, in considering the impact on testing decisions of the interplay between prevalence, sensitivity, and specificity as well as of other factors such as local resources, healthcare systems, and treatment strategies, the WHO has stated that IGRAs should not be used in high-prevalence settings [9]. Recent research does indicate, on the other hand, that IGRAs may be valuable in low-prevalence settings, where the focus on LTBI treatment renders highly specific tests the preferred option [10]. What remains to be established is whether decisions to use one IGRA over another in these lower-prevalence settings may be conducted in such a way as to optimize health outcomes and resource utilization.

This paper thus focuses on the interplay between sensitivity, specificity, and prevalence, independent of prevalence-test performance interactions. Specifically, we conducted two simple calculations – a hypothetical generic example, and one with two IGRA tests for LTBI screening – in settings of varying prevalence to quantify the impact of changing sensitivity and specificity on test outcomes. We assumed that sensitivity and specificity are independent of spectrum bias [11], and that spectrum bias is not present when transitioning between settings of varying prevalence. While this is somewhat artificial and not representative of reality, this simplifying assumption is made for purposes of demonstration.

## Methods

We estimated the number of true- and false-positive results that would occur when varying prevalence and test sensitivity and specificity. Inputs for the generic example were hypothetical and for demonstration purposes only, and those for the LTBI example were based on published reports and literature.

### Generic example

In the generic example, we predicted the number of true-positive and false-negative results that would occur with varying levels of test sensitivity and specificity, applied in four scenarios defined by disease prevalence rates of 20%, 40%, 60%, and 80%. Among a hypothetical cohort of 1,000 tested individuals, we calculated true-positive test results with test sensitivity at 50%, 60%, 70%, and 80% and false-negative results with specificity at 90%, 95%, 98%, and 99% (Tables 2 and 3).

**Table 2:** **True-positive results**^**a **^**in settings of varying prevalence and tests of varying sensitivity**

**Disease prevalence rate**	**Number of true-positive results**			
	**Sensitivity**			
	**50%**	**60%**	**70%**	**80%**
20%	100	120	140	160
40%	200	240	280	320
60%	300	360	420	480
80%	400	480	560	640

^a^ Predicted numbers based on a hypothetical population of 1,000 tested individuals.

**Table 3:** **False-positive results**^**a **^**in settings of varying prevalence and tests of varying specificity**

**Disease prevalence rate**	**Number of false-positive results**			
	**Specificity**			
	**90 %**	**95%**	**98%**	**99%**
20%	80	40	16	8
40%	60	30	12	6
60%	40	20	8	4
80%	20	10	4	2

^a^ Predicted numbers based on a hypothetical population of 1,000 tested individuals.

Results were calculated as:

$$\begin{array}{l} True\;Positive=Sensitivity\;*\;\mathrm{Pr}evalence\;*\;N\\ False\;Negative=(1-Specificity)\;*\;(1-\mathrm{Pr}evalence)\;*\;N \end{array}$$

where *N* = 1,000 individuals.

### LTBI testing example

We compared the consequences of implementing two IGRA tests – QuantiFERON^®^-TB Gold In-Tube (QFT-IT) and T-SPOT^®^.*TB* (T-Spot) – in settings of varying LTBI prevalence by applying the sensitivity and specificity of these tests to the generic example. We used estimates from a recently published meta-analysis to determine each test’s sensitivity and specificity as [12]:

$$\begin{array}{l} Sensitivity:QFT-IT=81\%,T-Spot=88\%\\ Specificity:QFT-IT=99\%,T-Spot=86\% \end{array}$$

These estimates represent results from 19 pooled QFT-IT studies and 17 pooled T-Spot studies for sensitivity as well as from five pooled QFT-IT studies and three pooled T-Spot studies for specificity. For all T-Spot analyses, a positive test threshold of ≥6 spots was used. Studies were published between 2006 and 2009 and included both developed and developing countries. Using these estimates in our calculations, switching from QFT-IT to T-Spot involves a 7% increase in test sensitivity and a 13% decrease in specificity.

The sensitivity and specificity differences between QFT-IT and T-Spot are primarily due to manufacturers’ applied positive test thresholds for each test [7,10,12,13]. In addition, such estimates of test operating characteristics are in fact highly population-dependent and vary with factors such as age, geographic region, exposure to other non-TB bacteria, stage and history of disease, and immunosuppression. However, we considered for this worked example fixed estimates of sensitivity and specificity, assuming that they remained stable within each country, and we used data from test manufacturers to ascertain that the positive test thresholds were equivalent for each test across countries. We therefore assumed in the calculations that prevalence was the only difference between settings [12,13].

We evaluated results when using QFT-IT and T-Spot in five countries chosen to represent a range of LTBI prevalence estimates from the WHO [4]: United States (5%), Mexico (29%), Brazil (39%), Thailand (47%), and Ivory Coast (55%). All calculations were developed and analyzed using Excel^®^ 2008 (^©^2007 Microsoft Corporation, Redmond, WA).

## Results

Table 2 demonstrates how varying both the sensitivity and prevalence affects the number of true positives. Similarly, Table 3 shows how changes in the specificity and prevalence affect the number of false positives. Table 2 shows that increasing sensitivity increased the number of true-positive results and that the absolute impact was greater in high-prevalence settings: every 10% increase in sensitivity produced 20 new true positives per thousand when prevalence was 20% but 80 new true positives when prevalence was 80%. On the other hand, Table 3 shows that the impact of specificity on false positives was greatest in the low-prevalence situation: compared with a “perfect” test, a 10% decrease in specificity produced 80 new false positives at a prevalence of 20% and 20 new false positives at a prevalence of 80%.

Table 4 shows the predicted number of true- and false-positive results and the ratio of false to true positives when switching from QFT-IT to T-Spot for LTBI screening in five countries with varying prevalences. When T-Spot (with a 7% greater sensitivity and 13% lower specificity than QFT-IT) was used, our results predicted that countries with a lower prevalence would have to pay a “price” of accepting more false positives for each true positive diagnosis than would countries with a higher prevalence. In the United States – with a 4.7% LTBI prevalence – switching tests would result in approximately 38 people receiving false-positive findings for each new true-positive diagnosis. On the other hand, when prevalence is approximately six times greater (at 28.8% in Mexico), the number of false-positive results per new true-positive diagnosis would decrease more than eight-fold to 4.6. With increasing prevalence, this false-positive to true-positive rate would continue to decline to 2.9:1 in Brazil, 2.1:1 in Thailand, and 1.5:1 in the Ivory Coast (with 38.7%, 46.7%, and 54.6% prevalence, respectively). Thus, at the most extreme comparison estimated here, when prevalence increased over 11-fold from 4.7% in the United States to 54.6% in Ivory Coast, the false- to true-positive ratio decreased more than 20-fold from 37.7 to 1.5.

**Table 4:** **True- and false-positive results**^**a **^**when changing from QFT-IT to T-Spot in screening for LTBI**

**Country**	**LTBI prevalence**^**b**^	**Increase in true positives**^**a,c**^	**Increase in false positives**^**a,c**^	**Increase in false positives for every true positive gained**
United States	4.70%	329	12,389	37.7
Mexico	28.82%	2,018	9,253	4.6
Brazil	38.74%	2,712	7,964	2.9
Thailand	46.74%	3,272	6,924	2.1
Ivory Coast	54.62%	3,823	5,900	1.5

LTBI, latent tuberculosis infection; QFT-IT, QuantiFERON-TB Gold In-Tube; T-Spot, T-SPOT.*TB*.

^a^ Based on a hypothetical population of 100,000 tested individuals.

^b ^*Source:* World Health Organization Global TB Database [4].

^c^ Sensitivity estimates: QFT-IT 81%, T-Spot 88%; specificity estimates: QFT-IT 99%, T-Spot 86%.

## Discussion

Our examples show that in addition to other factors, policymakers and clinicians should consider real sociodemographic factors such as prevalence when making choices between tests and when setting positive test thresholds in clinical practice as well as in policy guidelines. The interplay of sensitivity, specificity, and prevalence determines the balance of true and false results associated with a given diagnostic test strategy, and all three factors should be explicitly incorporated in evaluating testing programs. With any given test, as disease prevalence varies, the trade-off between the number of false-positive and false-negative results will vary, resulting in significant variations of health and economic consequences across settings.

Other investigators have considered the importance of incorporating prevalence in analyses of diagnostic tests. Sackett and colleagues demonstrated early on that for any given sensitivity and specificity the false-to-true positive ratio will decrease and the positive predictive value will increase with increasing prevalence [14]. It is also well acknowledged that sensitivity and specificity would likely change with varying prevalence, although this may be a manifestation of changing patient spectrum, with prevalence playing a secondary role [11]. The incorporation of prevalence along with sensitivity and specificity has further been described in conducting meta-analyses of diagnostic tests [15]. In addition, the issue of pre-test probability has been considered particularly relevant when using tests with an implicit or subjective threshold, where clinicians may move their subjective threshold in response to the perception of increased prevalence [16,17].

In the current analysis, we used screening for LTBI to demonstrate the importance of considering disease prevalence when evaluating such trade-offs in testing strategy decisions. We chose TB as an example because of its growing worldwide importance, its variations in prevalence (Figure 1), its diagnostic issues such as comorbidities and latent-versus-active disease, and the critical role of health systems and resources in determining optimal screening and treatment programs [3,4]. Although early detection and effective screening are critical to TB treatment and prevention, improvements in detection have recently slowed, with close to 40% of infections worldwide still not being properly detected or treated [5]. This slowing is in part due to the lower sensitivity and specificity of the standard TB skin test among certain populations [6-8] as well as the challenges in determining appropriate testing strategies in settings of highly varied levels of disease prevalence and resource constraints.

For example, in lower-burdened and higher-resourced countries such as the US, TB-control strategies target high-specificity LTBI screening and treatment to prevent later conversion to active TB. Such a strategy, however, is less common in higher-prevalence settings where resources are more often allocated towards treating those with active infection and especially in poorer settings where treatment costs may be more than double a household’s monthly income [18]. So although the introduction of newer tests such as QFT-IT and T-Spot can offer improved operating characteristics, improvements in outcomes depend on the establishment of testing strategies that are specific to each setting.

Thus, with the recent introduction of new tests for TB and the publication of WHO and FDA guidelines regarding their implementation, this analysis provides a timely demonstration that highly specific IGRA tests cause more harm and generate fewer benefits when used in high-prevalence countries, where there would be too many false negatives, too little treatment of diseased individuals, and more future illness and disease spread. In addition to confirming the WHO’s recommendations that IGRAs not be used in developing countries, our analysis also is useful to show that in making LTBI testing decisions *within* lower-prevalence developed countries, there may be benefits of using one IGRA test over another, depending on prevalence. The lower the prevalence, the more specific the test should be.

Our analysis is not only useful for making decisions between tests but also in determining setting-specific positive test thresholds. With the FDA’s 2009 decision to change the T-Spot cutoff for a positive result from six to eight spots [19,20], it actively weighed the sensitivity-specificity balance in the face of lower-prevalence TB in the US. This decision recognized that changing the threshold would decrease test sensitivity, yet it would increase specificity and result in improved outcomes for this setting. However, because of the inherent nature of the test, it is possible that this changed threshold may not increase specificity to the levels of the QFT-IT [10]. Therefore, if the revised T-Spot sensitivity and specificity values were known and included in the current analysis, the magnitude of differences in outcomes between the two tests would clearly diminish; however; the degree of decline is uncertain and is unlikely to be absolute. More research is needed to clearly determine the specificity of IGRAs in settings of varying prevalence, and in particular of the T-Spot assay with the revised US threshold.

Our TB example is a good demonstration of the issue of determining appropriate diagnostic testing strategies when the optimal sensitivity-specificity balance varies throughout the world. Consider, for example, two countries: one developed, the other not. Healthcare in the developed country is generally good, multi-drug resistant TB is relatively rare, and TB prevalence is relatively low. In the developing country, healthcare access is more limited, resistant TB is more common, and TB prevalence is higher.

An LTBI test such as T-Spot that offers a significant increase in sensitivity – compared with QFT-IT – at the cost of a 13% decrease in specificity may be valued differently in the two countries. The developed country may find that the 7% increase in early case detection benefits too few people to justify the high burden of false positives. The developing country may find that with higher disease prevalence, the greater increase in early detection is worth the increased treatment of false-positive cases, especially given the poorer access to medical services. This is not to say that the trade-off is not worthwhile in the developed country or that it is worthwhile in the developing country. Resources and local priorities and values should determine that. Rather, one should not expect the trade-off to be similar in different areas; indeed, it may differ by orders of magnitude as prevalence varies.

Despite this differential impact between settings, testing decisions do not always consider specific populations and disease characteristics, and like those for QFT-IT and T-Spot, positive-result thresholds are usually set at a global level by manufacturers and applied consistently across countries [12,13]. Given that the prevalence of many diseases varies worldwide, encouraging policymakers to explicitly incorporate disease prevalence in their testing decisions and allowing them to choose setting-specific thresholds – or to choose from a menu of possible choices – could increase the value of a given test by optimizing test performance and improving health and economic outcomes.

Tuberculosis is a good example for demonstrating the impact of prevalence in decisions regarding positive thresholds and test strategies because of issues such as the challenges of estimating accurate test operating characteristics, the varying disease prevalence, and the differences between active and latent infection. Although such issues apply when testing for any disease, they must be taken into account when interpreting the implications of our analysis. For example, the impact of incorrect LTBI diagnoses can be particularly difficult to estimate because of low treatment compliance and the challenge of estimating the impact of delayed diagnoses. This analysis also ignores other issues involved in testing for the less-prevalent active TB [6-8].

In addition, test sensitivity and specificity and the impact of prevalence are not the only determinants of a test’s usefulness, and decisions regarding positive test thresholds and test usefulness in different settings must consider a multitude of factors. To name but a few: variation in estimates of test sensitivity and specificity (e.g., as determined by factors such as study methodology); balance of risks and benefits; reason for testing (screening or diagnosis); population-specific geography and demographics; patient preference; and patient values for different outcomes (e.g., associated with culture). Testing programs may maximize benefit, minimize risk, and successfully prevent and treat disease only when all such factors are considered. Although the examples discussed herein come from only one disease (TB), this should not be considered a limitation of the study. Rather, this analysis demonstrates an epidemiologic principle that holds true for any disease, even though the magnitude of effect will vary from one disease to another.

## Conclusions

No matter what the sensitivity and specificity of a test are, the prevalence determines the absolute numbers of missed cases and over-treated non-cases. Authors of primary studies and systematic reviews of diagnostic accuracy could be more aware of this issue. Testing policies should specifically address each setting’s disease and population characteristics, and sensitivity and specificity should be evaluated as a function of all relevant criteria that include disease prevalence and positive thresholds.

Future research should evaluate the benefit-risk trade-offs involved in incorporating new and standard tests, at varying positive test thresholds, and in high- and low-prevalence settings. Evaluating the trade-offs between true and false positives can aid decision makers in deciding between tests of varying sensitivity and specificity, in determining the optimal threshold for a positive test, and in ultimately optimizing disease-related outcomes in different global settings.

## Competing interests

AC serves as a consultant for Qiagen/Cellestis, the manufacturer of QuantiFERON-TB Gold In-Tube. TB and TG have no competing interests to declare.

## Authors’ contributions

AC participated in the design and coordination of the study and helped to draft the manuscript. TB participated in the design and coordination of the study, conducted literature searches, collected data, performed analyses, and drafted the manuscript. TG conceived of the study, participated in its design and coordination, and helped to draft the manuscript. All authors read and approved the final manuscript.
